# Oral Squamous Papilloma Mimicking Oral Verrucous Leukoplakia

**DOI:** 10.7759/cureus.31739

**Published:** 2022-11-21

**Authors:** Houda Tebcherany, Anupama Grandhi, Ahmed Khocht

**Affiliations:** 1 General Dentistry, Loma Linda University School of Dentistry, Loma Linda, USA; 2 Oral & Maxillofacial Surgery, Loma Linda University School of Dentistry, Loma Linda, USA; 3 Periodontics, Loma Linda University School of Dentistry, Loma Linda, USA

**Keywords:** human papilloma virus, verrucous leukoplakia, squamous papilloma, oral mucosa, oral cavity

## Abstract

The human papillomavirus (HPV) is a non-enveloped DNA virus that causes a variety of skin and mucosal lesions. This report reviews a likely HPV-related lesion of oral squamous cell papilloma clinically mimicking oral verrucous leukoplakia. A 71‐year‐old white male presented with a raised white lesion on the palatal mucosa. It felt hard on palpation and had a sessile fixed base, and a rough verrucous surface. The lesion was fully excised. Histopathology showed short, thin, fingerlike projections lined by stratified squamous epithelium with thin central connective tissue cores. The epithelial superficial layers demonstrated focal koilocytotic changes suggestive of an HPV infection. High-risk HPV-related lesions have the potential to turn malignant. Early diagnosis and management are critical to preventing serious complications.

## Introduction

Human papillomavirus (HPV) is a non-enveloped DNA virus belonging to the Papillomaviridae family [1]. Several hundred genotypes of HPV have been identified in humans. HPV is involved in the etiology of a variety of skin and mucosal lesions and is the most common sexually transmitted infection, with more than 3 million cases reported per year in the United States [2]. Many individuals with HPV don't develop any symptoms but can still infect others through sexual contact. HPV infections cause small benign tumors (e.g., papilloma, warts, or verruca), with some lesions carrying a risk of becoming cancerous. In the oral cavity, common HPV-related lesions include squamous papilloma, verruca vulgaris, condyloma acuminatum, and focal epithelial hyperplasia [3].

This report describes an interesting case of oral squamous papilloma mimicking oral verrucous leukoplakia on the palatal mucosa of an adult male patient.

## Case presentation

**Figure 1:**
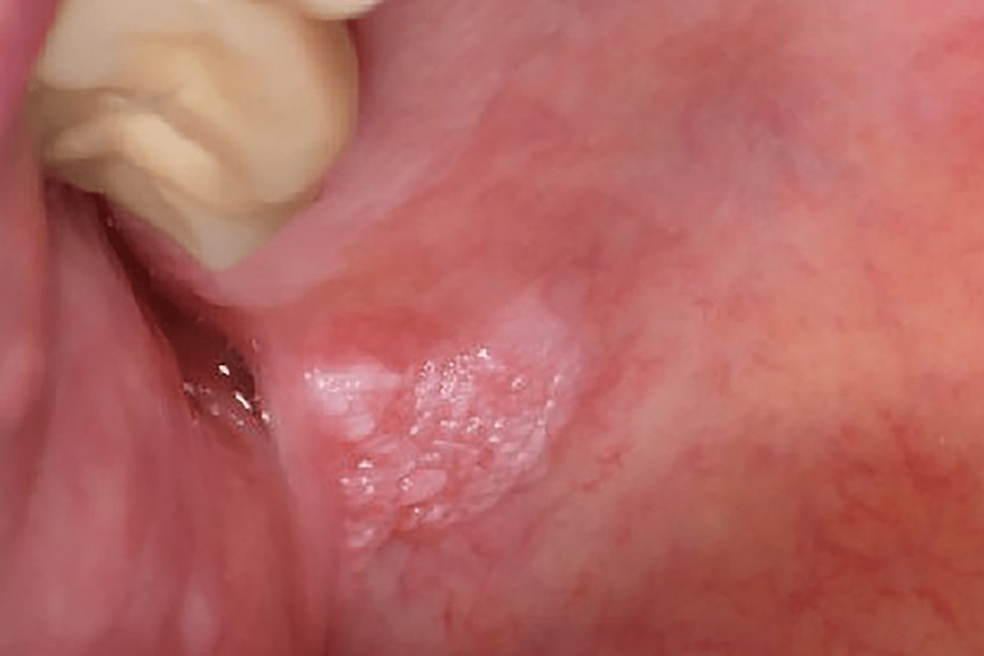
Clinical presentation Verrucous lesion at the junction of the soft and hard palate on the right side.

A 71‐year‐old white male with a history of hypertension presented to Loma Linda University School of Dentistry Wellness Center with the chief complaint of a white lesion in the roof of the mouth on the right side. The patient was aware of the lesion for several months. He stated that his tongue rubbed against the lesion’s rough surface, and it felt like a scratch. The lesion was not associated with any discomfort. A clinical examination showed an irregularly outlined lesion at the junction of the soft and hard palate on the right side (Figure 1). 

The lesion was a raised rough pink to white plaque with a verrucous surface and somewhat ill-defined margins. It measured 6 by 6 millimeters. It felt hard on palpation and had a sessile fixed base. A slight increase in vascularity was visible in the mucosa around the base of the lesion.

There were no other lesions in the oral cavity or extra-orally. No cervical lymph nodes were palpable. The patient had no history of smoking or alcohol consumption. A provisional diagnosis of oral verrucous leukoplakia was made. The differential diagnosis includes verruca vulgaris, squamous papilloma, and condyloma acuminatum [4].

**Figure 2:**
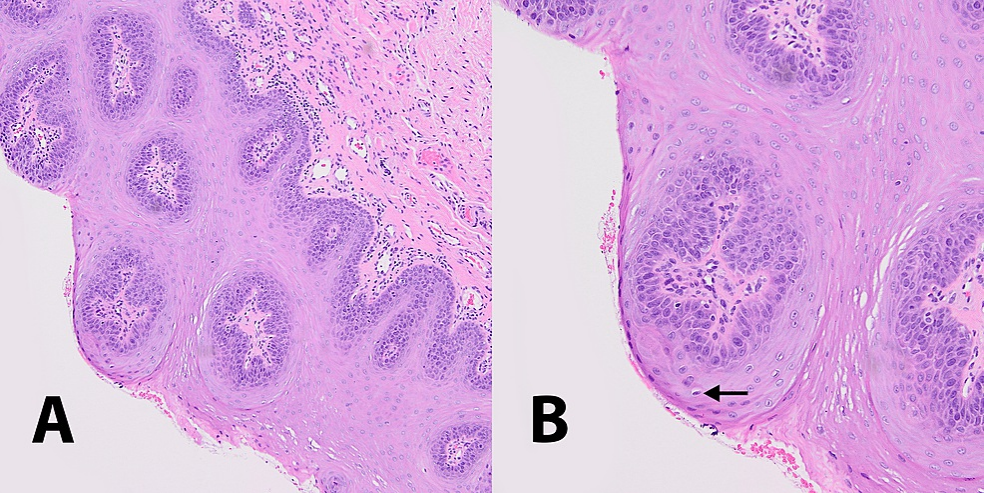
Histopathologic characteristics A. Tangential section showing epithelial proliferation of individual papillae centered around connective tissue cores (magnification 100x). B. Superficial layers of the epithelium showing focal koilocytotic changes indicated by arrow (magnification 200x).

Under local anesthesia, the lesion was fully excised with a scalpel and sent for histopathological examination. Histopathological examination showed short, thin, and fingerlike projections lined by stratified squamous epithelium with thin central connective tissue cores (Figure 2A). The epithelium displayed acanthosis, parakeratosis, and papillomatosis. Superficial layers of the epithelium demonstrated focal koilocytotic changes with perinuclear cytoplasmic halos suggestive of HPV infection (Figure 2B). No dysplasia was identified.

Based on the clinical and histologic features, a diagnosis of oral squamous papilloma was made. Clinical follow‐up at six months postoperatively showed complete tissue healing with no signs of recurrence.

## Discussion

The case presented here reviewed the clinical appearance and histopathology of a squamous papilloma lesion on the palatal mucosa of an adult male and discussed how it was managed. The clinical appearance showed a sessile leukoplakic white lesion with a verrucous surface resembling oral verrucous leukoplakia. However, the histopathology was more consistent with squamous papilloma. Squamous papilloma is the most common benign epithelial neoplasm of the oral cavity [5]. It is a benign, usually pedunculated, epithelial enlargement characterized by small fingerlike projections, resulting in a rough cauliflower surface. It arises from the surface stratified squamous epithelium and does not invade underlying tissue. Although malignant transformation of oral squamous papilloma is rare, elderly patients have a three-fold increased carcinoma risk compared with nonelderly patients [6]. Excisional biopsy is the treatment of choice, and recurrence is unlikely.

The presence of koilocytotic changes within the epithelial cells in the presented case is suggestive of HPV involvement. HPV-6 and HPV-11 are commonly associated with squamous papilloma [7]. The virus particles invade keratinocytes with consequent epithelial proliferation and nodule formation. Patient habits may play a role in promoting HPV infections and in the subsequent neoplastic changes in the infected tissues. Cigarette smoking and alcohol abuse can modify tissue permeability and promote HPV tissue invasion [8]. Furthermore, the oral microbiota may adversely influence HPV infections [9].

In the presented case, although the epithelial koilocytotic changes noted were suggestive of HPV involvement, viral genetic typing was not done. Since the lesion was fully excised and no dysplasia or neoplasia was present, we felt no additional testing was necessary.

Other similar oral HPV-related lesions include verruca vulgaris and condyloma acuminatum. Oral verruca vulgaris (wart) is an HPV-related benign proliferation of the stratified squamous epithelium. HPV-2 and HPV-4 are the genotypes most related to oral verruca vulgaris [10]. It is more common in males [11]. It is typically seen extraoral on hands, feet, toes, and fingers [11]. While HPV-related oral lesions are fairly common, verruca vulgaris intraorally is uncommon [12]. Oral lesions are typically found in children but can manifest in any age group. A few cases were previously reported in adults on the tongue [13], hard palate [12] and the buccal mucosa [4]. Some lesions show a cauliflower surface while others show finger‐like projections. The color of the lesion is dependent on the degree of keratinization, coral pink if non-keratinized and white if keratinized. In the presented case, the lack of a strong epithelial granular cell layer ruled out a diagnosis of verruca vulgaris.

Condyloma acuminatum lesions are usually found in the genital area and are considered a sexually transmitted disease [14]. Condyloma acuminatum lesions are also associated with HPV-6, HPV-11, HPV-16 and HPV-18 [7]. Oral lesions arise from oral sex. They are similar in appearance to squamous papilloma but somewhat larger in size and tend to be deep rooted. They are usually present in groups of multiple lesions on the labial mucosa, soft palate, and tongue. Recommended treatment is also surgical excision.

Some leukoplakic lesions may also present with a verrucous appearance. Oral verrucous leukoplakia is a rare form of oral leukoplakia with a high potential for malignant transformation exceeding 70% [15,16]. Women are more commonly affected than men and the mean age at the time of diagnosis is over 60 years [15,16]. The buccal mucosa and tongue are the most affected sites; palatal mucosa, alveolar mucosa, gingiva, floor of mouth, and lip are less likely to be affected [15-17]. Oral verrucous leukoplakia usually simultaneously involves multiple oral sites [15-17]. It is characterized by a high recurrence rate and histological progression to squamous cell carcinoma or verrucous carcinoma [16,17]. Histopathologic features of oral verrucous leukoplakia vary from entirely benign hyperkeratosis to frankly malignant features, with areas of squamous cell carcinoma [15,16]. Because of the lack of distinctive histological criteria, the diagnosis of oral verrucous leukoplakia is based on a combination of clinicopathological features. In the present case, although the clinical appearance of the lesion mimicked an oral verrucous leukoplakia, the histologic features did not correlate with the clinical presentation.

Several studies have investigated the role of HPV in oral verrucous leukoplakia [17-19]. The degree of involvement of HPV with oral verrucous leukoplakia varies greatly between reported cases [18,19]. A relatively large study evaluating HPV involvement in verrucous and conventional leukoplakia reported HPV DNA rates of 24.1% and 25.5%, respectively [19]. Thus, the association between HPV and oral verrucous leukoplakia seems to be ambiguous. HPV genotypes related to malignant and premalignant oral verrucous leukoplakia lesions in the oral cavity are mainly HPV-16 and HPV-18 [7].

Various treatment modalities have been proposed to manage HPV-related lesions including topical agents such as salicylic acid and vitamin A. However, surgical excision with adequate margins is the treatment of choice. Scalpel excision, as performed in the presented case, is the most commonly used modality to excise these lesions. Cryotherapy and laser surgery have also been shown to be effective in treating such lesions [20]. Laser management offers multiple advantages including less intraoperative bleeding, minimal damage to adjacent tissue, delayed acute inflammatory reaction, and reduced myofibroblast activity, leading to reduced wound contraction and scarring [20].

## Conclusions

HPV-related oral infections are common in the oral cavity, and high-risk HPV can cause cancer. Herein, we presented a case of HPV-related oral squamous papilloma on the palatal mucosa which mimicked clinical features of oral verrucous leukoplakia. We discussed major differential points that clinicians and pathologists should consider during diagnosis of oral papillary lesions. Early diagnosis and management are critical to preventing serious complications. Diagnosis is essentially based on clinical characteristics, but histopathologic confirmation is recommended.
